# Vasculotoxic and Proinflammatory Effects of Plasma Heme: Cell Signaling and Cytoprotective Responses

**DOI:** 10.1155/2013/831596

**Published:** 2013

**Authors:** John D. Belcher, Karl A. Nath, Gregory M. Vercellotti

**Affiliations:** 1 Division of Hematology, Oncology and Transplantation, Vascular Biology Center, Department of Medicine, University of Minnesota Medical School, 420 Delaware Street SE, Minneapolis, MN 55455, USA; 2 Division of Nephrology and Hypertension, Mayo Clinic, Rochester, MN, USA

## Abstract

The proinfammatory vasculotoxic effects of intravascular hemolysis are modulated by plasma hemoglobin and heme clearance via the haptoglobin/CD163 system and the hemopexin/CD91 system, respectively, and detoxification through the heme oxygenase/ferritin system. However, sudden or excessive hemolysis can overwhelm these protective systems leading to heme interacting with cells of the vasculature. Heme presents a damage-associated molecular pattern to the innate immune system. Heme is an extracellular inflammatory signaling molecule with strict binding specificity for TLR4 on monocyte/macrophages, endothelial, and other cells. The resulting TLR4 signaling cascade rapidly leads to intracellular oxidative stress and an inflammatory response. Heme also induces a cytoprotective response that includes Nrf2 responsive genes such as heme oxygenase-1, ferritin, haptoglobin, hemopexin, and other antioxidant response genes. It is the balance between the pro-inflammatory/vasculotoxic effects of plasma hemoglobin/heme and the cytoprotective responses that ultimately determines the pathophysiologic outcome in patients.

## 1. Introduction

When hemoglobin (Hb) is released from red blood cells (RBCs) into plasma, it has the potential to release free heme that can trigger severe oxidative, proinflammatory, and pro-thrombotic injury. Heme has several proinflammatory activities, including leukocyte activation and migration, upregulation of adhesion molecules, reactive oxygen species (ROS) production, and induction of cytokine and chemokine expression [1–4]. Organisms have evolved intricate systems to defend against free heme. The term “free” heme will be used loosely in this review, as heme is amphipathic, mostly insoluble in aqueous solutions at neutral pH, and likely bound to proteins and/or lipids *in vivo*. This review will focus on the proinflammatory and anti-inflammatory molecular signaling events that are activated by cells in response to free heme.

## 2. Hemolysis and Plasma Defenses

Intravascular hemolysis releases Hb from an antioxidant rich environment inside the RBC into the plasma. Once free in plasma the Hb tetramer is in equilibrium with the α/β Hb dimer favored at low plasma Hb concentrations. The Hb dimers bind to haptoglobin which can safely carry Hb to CD163 receptors on macrophages where the Hb is degraded [5–7]. However, during massive or chronic hemolysis, the haptoglobin/CD163 system can be overwhelmed, leaving free Hb in plasma. When not bound to haptoglobin, oxyHb in plasma can react rapidly with nitric oxide (NO) to form nitrate (NO_3_^−^) and ferric Hb (metHb). To illustrate this point, steady state metHb levels can reach as high as 5% in humans inhaling 80ppm NO for 4 hours [8]. However, steady-state metHb levels do not accurately reflect the kinetic throughput of metHb in plasma because metHb is unstable and releases ferric heme (hemin) from the globin chain [9]. The released hemin binds to plasma hemopexin (Kd < 10^−12^ M), albumin (Kd ~ 10^−8^ M) or intercalates into plasma lipoproteins (Kd 10^−10^M to 10^−11^M) or cell membranes because of its amphipathic structure [10, 11]. Because of its high binding affinity, hemopexin is the first line of defense against hemin released from metHb. Hemopexin delivers the bulk of hemin to CD91 receptors on hepatocytes where it is endocytosed and degraded [12].

## 3. Heme Stimulation of TLR4 Signaling Is Proinflammatory

Like haptoglobin/CD163, the hemopexin/CD91 system can be overwhelmed during periods of excessive hemolysis. In hemopexin null mice, there is enhanced clearance of heme by nonhepatic tissues including the vessel wall [13]. Recently, heme has been shown to be an extracellular inflammatory signaling molecule in macrophages with strict binding specificity for toll-like receptor-4 (TLR4) [4]. These findings imply that heme is a damage-associated molecular pattern (DAMP) molecule. Signaling of heme through TLR4 is distinct from the signaling of bacterial lipopolysaccharide (LPS) through TLR4 [4]. Heme activation of TLR4 is remarkably stringent and requires the iron moiety [4, 14]. Unlike its analogs or precursors, heme induces macrophage tumor necrosis factor-alpha (TNF-α) secretion that is dependent on TLR4 and its adaptor molecules MYD88 and CD14 [4]. By binding hemin, hemopexin thwarts hemin-mediated TLR4 signaling and thereby prevents the proinflammatory effects of hemolysis [13, 14].

Endothelial cells also express TLR4 [15–18]. In endothelium, hemin-, or LPS-mediated TLR4 signaling stimulates two parallel proinflammatory/prothrombotic pathways: (1) Weibel-Palade Body (WPB) degranulation [14] and (2) nuclear factor-kappa B (NF-κB) activation [14, 19]. WPB degranulation occurs within 5 minutes of hemin (or LPS) stimulation of TLR4. WPB degranulation releases numerous vasoactive proteins including von Willebrand Factor, P-selectin, endothelin-1, endothelin-converting enzyme, interleukin-8, tissue-type plasminogen activator, eotaxin-3, angiopoietin-2, osteoprotegerin, and calcitonin gene-related peptide [20]. Hemin/TLR4-mediated degranulation of WPBs on the endothelial cell surface triggers rapid proinflammatory, prothrombotic, and vasoconstrictive responses in the vasculature.

In addition to WPB degranulation, hemin- or LPS-mediated TLR4 signaling leads to NF-κB activation and the transcription of proinflammatory genes [19]. Inhibition of TLR4 signaling or knockout of the TLR4 gene completely abrogates hemin-stimulated WPB degranulation and NF-κB activation in endothelial cells [14]. Heme amplifies LPS-induced TLR4 signaling [21], which may explain why hemolysis and hemoglobinuria are associated with increased mortality in septic patients [22, 23].

Hemin also has procoagulant effects on endothelium (von Willebrand factor and tissue factor expression) [14, 24] that could be explained by hemin-mediated TLR4 signaling [14]. Moreover, hemin activation of TLR4 signaling in monocytes and platelets could potentially induce thrombosis via tissue factor expression on monocytes (unpublished data) and platelet activation.

Hemin also induces oxidative stress in cells. Much of this has been attributed to iron-catalyzed reactions [25]. However, there is evidence that LPS activated TLR4 in endothelial cells interacts with NADPH oxidase 4 (Nox4), a protein related to gp91^phox^ (Nox2) of phagocytic cells and that Nox4 activity is required for TLR4-mediated NF-κB activation [26]. The Nox protein generates superoxide (O_2_^−^) by transferring electrons from NADPH to O_2_. These superoxide free radicals are rapidly converted to H_2_O_2_ and O_2_ in cells. Thus, heme-mediated TLR4/NADPH oxidase signaling may explain much of the cellular oxidative stress that occurs during hemolysis.

## 4. Cytoprotection by Heme Oxygenase

Once heme enters the cell membrane, the next line of defense is heme oxygenase (HO). HO degrades heme into carbon monoxide (CO), biliverdin, and iron (Fe^2+^). There are two isoforms of HO, HO-1 and HO-2 [27]. HO-1 is highly inducible by a large number of oxidative stressors including heme. In normal healthy tissues, not involved in ongoing RBC destruction, HO-1 levels are low [27]. In tissues such as the kidney, HO-2, a constitutive enzyme, is an important first responder to intracellular heme. HO-2 null mice (HO-2^−/−^), compared with HO-2^+/+^ mice, exhibit greater renal dysfunction and histologic injury when administered Hb [28]. However, within 3 hours, HO-1 expression quickly surpasses HO-2 and HO-1 becomes the principle cytoprotectant to subsequent exposures to heme [29]. The mechanisms controlling HO-1 induction are complex, cell specific, and tightly regulated by transcription. A large number of kinases (e.g., mitogen-activated protein kinases (MAPKs), protein kinase C (PKC), and phosphatidylinositol 3-kinase (PI3 K)/protein kinase B (Akt)) and transcription factors (e.g., NF-κB, activator protein-1 (AP-1), nuclear factor E2-related factor 2 (Nrf2), biliverdin reductase (BVR) and BTB and CNC homologue 1 (Bach1)) are involved in regulating HO-1 expression [30]. Nrf2 appears to be an especially important regulator of HO-1 induction in response to oxidative stress and heme [31, 32]. Nrf2 normally resides in the cytoplasm bound to an inhibitor protein: kelch-like ECH-associated protein-1 (Keap1). Keap1 expedites the ubiquitination and proteolysis of Nrf2. Oxidation of cystine residues to cysteine (i.e., disulfide cross-linking) on Keap1 releases Nrf2, prolongs Nrf2 half-life, and allows Nrf2 transport to the nucleus [33]. Molecular signaling by the PI3 K/Akt pathway also plays an important role in Nrf2 activation. Activated Nrf2 translocates to the nucleus and binds to antioxidant response elements (ARE), which promotes the transcription of a wide variety of antioxidant/anti-inflammatory genes including HO-1, ferritin, haptoglobin, and hemopexin. Heme also binds to the transcriptional repressor Bach1 in the nucleus. Bach-1 is a member of the bZIP transcription factor family that serves as a repressor of HO-1 transcription through its higher binding affinity for multiple Maf recognition element (MARE) regions compared with Nrf2 [34]. When Bach1 forms a heterodimer with Maf, it binds to MARE regions and represses HO-1 transcription. Heme binding to Bach1 releases Bach1/Maf repression allowing Nrf2 binding and HO-1 transcription.

## 5. CO Mimics HO-1 Cytoprotection

The cytoprotective properties of HO-1 are mimicked by CO, which is released from heme by the HO reaction. CO gas is produced by HO-mediated opening of the heme ring. CO is a colorless, odorless gas that has traditionally been considered a dangerous poison. This toxicity is in part due to its high affinity for Hb (234X greater than O_2_), altering O_2_ transport and delivery [35]. CO also can interact with other heme proteins. However, like many other compounds this gas encompasses both a toxic and a therapeutic range [36]. At low concentrations, CO is a potent mediator of cell protection and has a number of properties that make it an attractive therapeutic option for treating hemolytic diseases. CO mimics many of the protective effects of HO-1, as well as some of the functions of NO. [3, 36, 37] Like NO, CO activates the heme protein guanylate cyclase, inhibits platelet activation and aggregation, and has a possible role as a neurotransmitter [3, 37]. Exogenous inhaled CO, at approximately 250 parts per million (ppm), and in some studies as low as 10 ppm [38], reduces inflammatory responses in several models of oxidant injury in similar ways to HO-1 overexpression [37]. CO interacts with signal transduction pathways, inhibits proinflammatory genes, and augments anti-inflammatory cytokines [3, 37, 39, 40]. Specifically, it selectively activates cytoprotective p38 MAPK and Akt signaling pathways in a guanylate cyclase-independent manner [37, 39]. CO also inhibits proliferation of vascular smooth muscle cells and has antiapoptotic effects on cells [3, 37].

CO induces HO-1 expression by its action on Nrf2, thus amplifying HO-1 expression [41, 42]. CO initially acts via inhibition of cytochrome *c* oxidase in the mitochondrial electron transport chain leading to the generation of low levels of O_2_^−^ and subsequently hydrogen peroxide (H_2_O_2_) that initiates the ensuing adaptive signaling [36]. Inhaled CO in mice or treatment of keratinocytes with H_2_O_2_ induces the phosphorylation/activation of p38 MAPK and Akt [43, 44]. Analysis using specific inhibitors of p38 MAPK and Akt has demonstrated that only Akt activation is involved in HO-1 and Nrf2 expression [44]. In addition, PI3 K and PKC inhibitors suppressed Akt phosphorylation, Nrf2 activation, and HO-1 expression [44]. Additional studies in knockout animals are warranted to further define the molecular signaling pathways responsible for upregulation of HO-1 by CO.

Thus CO induces an antioxidant (Nrf2 responsive genes) and anti-inflammatory (e.g., NF-κB suppression, HO-1 and interleukin-10 upregulation) response. In addition, CO may inhibit TLR4 signal transduction by enhancing the interaction of TLR4 with caveolin-1 [45] and by downregulating TLR4 expression [46].

## 6. Biliverdin Cytoprotection

Biliverdin is produced by the HO reaction with heme. Biliverdin reductase (BVR) catalyzes the reduction of biliverdin to bilirubin. BVR is expressed on the exterior of the plasma membrane where it quickly converts biliverdin to bilirubin [47]. The enzymatic conversion of biliverdin to bilirubin by BVR initiates a signaling cascade that results in a rapid increase in phosphorylation of Akt, leading to cytoprotection, due in part to upregulation of interleukin-10 expression [47]. In addition, phosphorylated Akt phosphorylates endothelial nitric oxide synthase (eNOS) in endothelial cells leading to S-nitrosylation of BVR [47]. S-nitrosylation of BVR leads to nuclear translocation, where BVR binds to AP-1 sites in the TLR4 promoter and blocks transcription of TLR4 [47]. In addition, human BVR is a Ser/Tr/Tyr-kinase and upstream activator of PKC and the insulin/insulin growth factor-1 pathways [48]. Thus like CO, biliverdin reduction to bilirubin by BVR regulates vital homeostatic signaling pathways in response to hemolysis.

## 7. Ferritin Heavy Chain (FHC) Cytoprotection

The induction of HO-1 is accompanied by the induction of ferritin [49]. Iron (Fe^2+^), released during the HO reaction, induces the translation of ferritin [50]. Labile cellular iron stimulates the translation of ferritin mRNA through interaction between a cytoplasmic iron regulatory protein (IRP) and a conserved nucleotide iron responsive element (IRE) present in the 5′ noncoding region of all ferritin mRNAs. The IRE forms a stem-loop structure and when the supply of iron to the cells is inadequate, the IRP is bound to the IRE and suppresses ferritin synthesis [51]. Ferritins are comprised of various ratios of heavy and light chains that form a protein shell surrounding an iron core. Ferritin is cytoprotective in cells, by its capacity to bind 4,500 iron molecules and through its FHC ferroxidase activity [52], which oxidizes redox active Fe^2+^ to Fe^3+^ for safe (redox inactive) storage in the core of the ferritin complex. FHC is protective against heme-mediated oxidative injury to endothelial cells *in vitro* [49]. FHC mutants lacking ferroxidase activity are not cytoprotective against heme-mediated oxidative injury. Overexpression of FHC protects tissues from ischemia-reperfusion injury [53], antagonizes TNFα-mediated apoptosis [54], protects cells from UV-radiation damage [55], prevents 1-methyl-4-phenyl-1,2,3,6-tetrahydropyridine-(MPTP-) induced neurotoxicity [56], and protects HeLa cells from H_2_O_2_ toxicity [57]. Nuclear FHC may play an important role in cytoprotection. Identification of a DNA binding motif for FHC raises the novel possibility of a role for FHC as a conventional transcription factor [58]. Nuclear FHC has been reported to incorporate into DNA and to protect DNA from UV and oxidative damage. FHC also binds with nuclear death domain-associated protein to inhibit DAXX-mediated apoptosis [59, 60].

## 8. HO-1 and Sickle Cell Disease

Sickle cell disease is an archetypal example of a chronic hemolytic disease. An inherited mutation, the amino acid glutamic acid is replaced with the amino acid valine at position 6 in β-globin (Glu6Val). The resulting Hb-S polymerizes in the de-oxygenated state forming long rigid molecules that stick together. The polymerization of Hb-S leads to various shape changes in RBC including an elongated sickle or crescent shape, release of heme into RBC membranes, oxidation of RBC lipids, and ultimately both intravascular and extravascular hemolysis [61]. Excessive release of Hb-S from sickle RBC into plasma can lead to the depletion of plasma haptoglobin, hemopexin and NO [62, 63], deposition of heme iron in cells and a proinflammatory/prothrombotic phenotype that promotes episodic painful vasoocclusive crises leading to ischemia/reperfusion injury and organ infarction.

**Figure 1:**
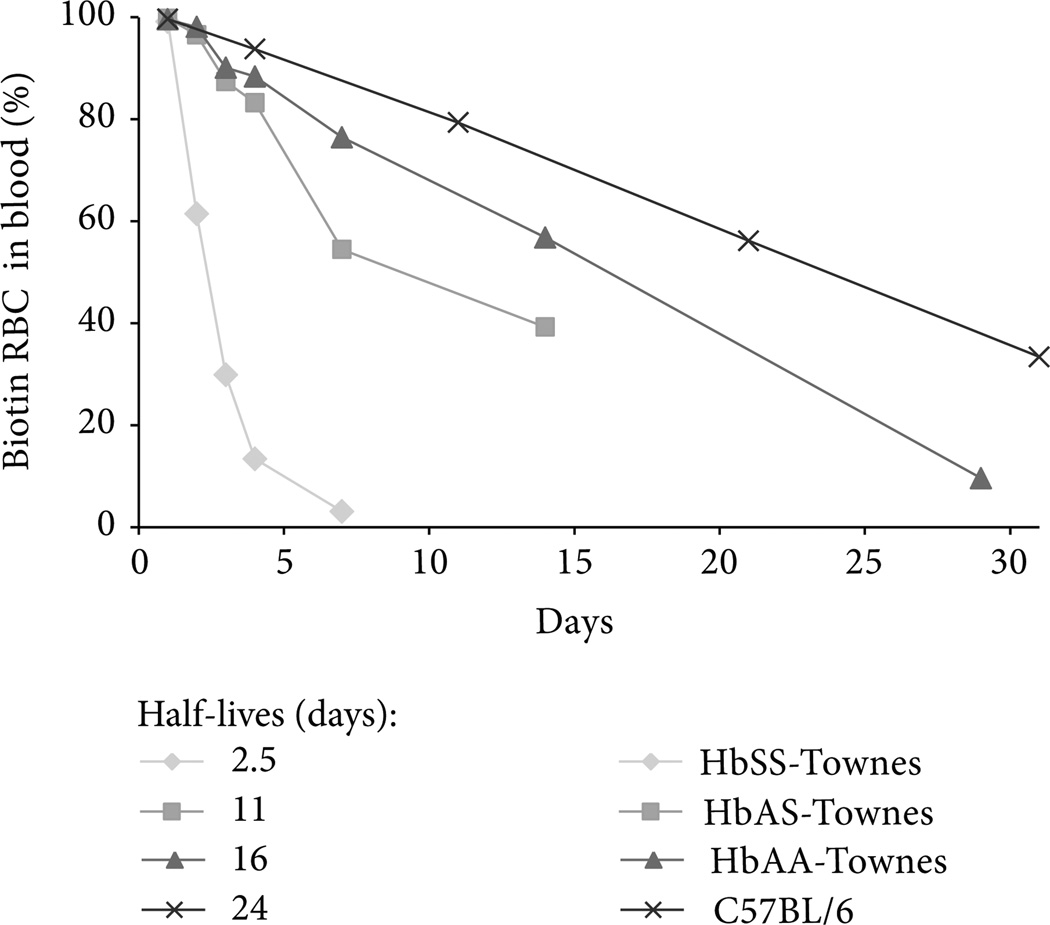
Red blood cell survival in C57BL/6, HbAA-, HbAS-, and HbSS-Townes mice. RBC half-lives were measured in C57BL/6, HbAA-, HbAS-, and HbSS-Townes mice (*n* = 5–7 mice/group). Values are means; standard deviations have been omitted for clarity, but coefficients of variation were less than 10%. The half-lives of circulating RBCs were measured by biotinylating RBCs by tail vein injection of 150 µL of 30 mg/mL sulfo-N-hydroxysuccinimide-biotin (Thermo Scientific, Rockford, IL, USA). Biotinylated RBCs were labeled with streptavidin-APC and the percentages of labeled and unlabeled RBC were measured by flow cytometry. RBC half-lives were estimated by interpolating between data points on either side of 50% survival. Half-lives for each mouse model are listed below the figure.

**Figure 2:**
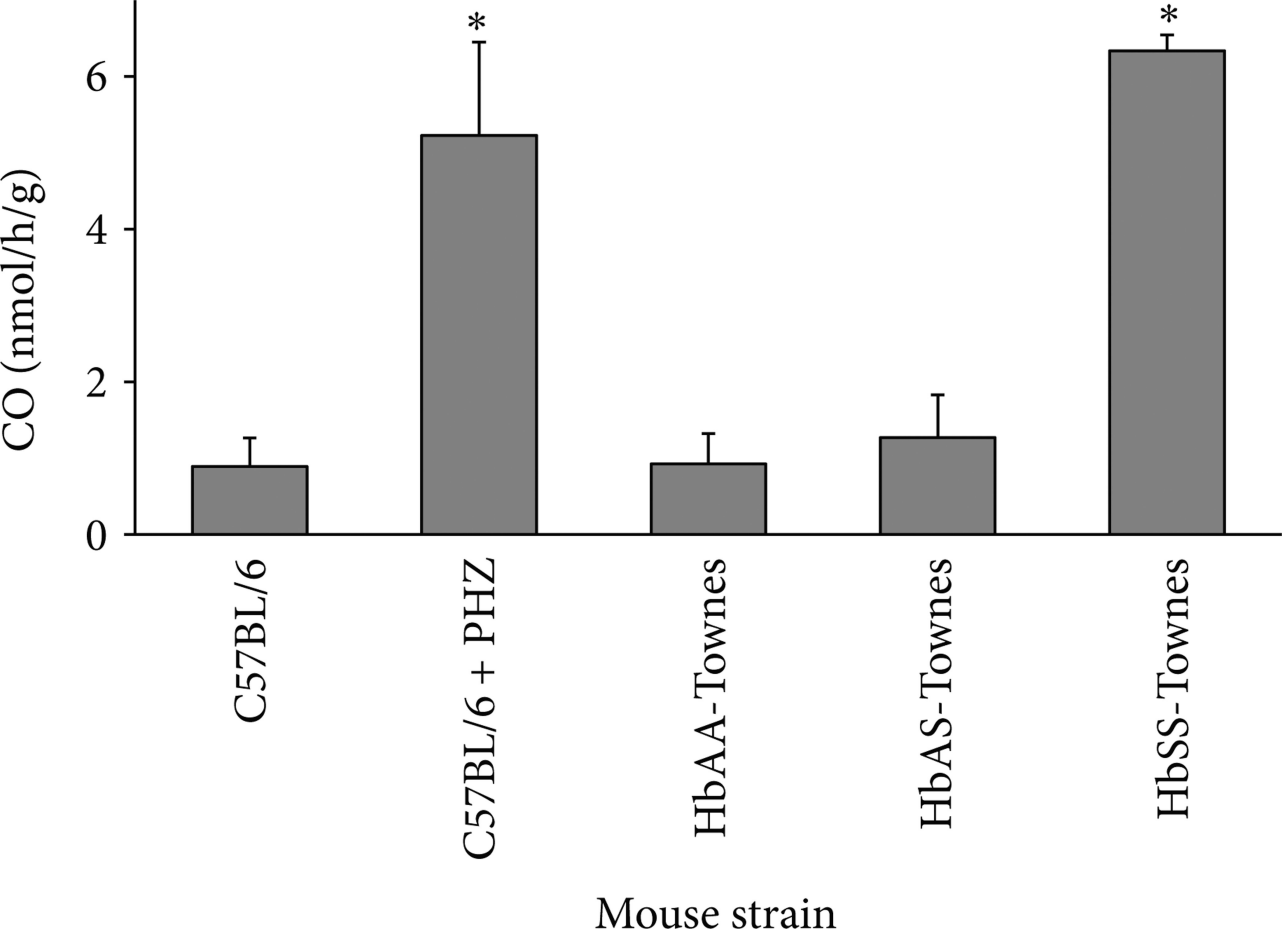
Endogenous CO production in mouse models of SCD. Exhaled CO was collected for 3 hours from C57BL/6, HbAA-, HbAS-, and HbSS-Townes mice (*n* = 5–7 mice/group). C57BL/6 mice injected with phenylhydrazine, an hemolysis inducing drug, 48 and 24 hours before collection of exhaled CO, served as positive controls for breath CO measurements. Exhaled CO was 5.9-fold higher in C57BL/6 mice injected with phenylhydrazine (*n* = 4) than untreated C57BL/6 mice (*n* = 12, * *P* < 0.001). Exhaled CO levels were not significantly higher than untreated C57BL/6 controls in the HbAA- and Hb-AS-Townes (*n* = 4) models. Exhaled CO levels were highest in HbSS-Townes mice (*n* = 4), 6.9-fold higher than HbAA-Townes mice (*n*= 4, * *P* < 0.001). Values are means ± SD.

Sickle cell disease patients in crisis-free steady state are experiencing unrelenting chronic hemolysis. The rate of hemolysis can be determined by measuring the half-life of RBC in patients or transgenic mouse models of sickle cell disease (Figure 1). The β^murine^ knock-out/β^S^ knock-in mouse model developed by the Townes laboratory [64] can be used to illustrate this point. RBC half-lives were measured in separate cohorts of HbAA-Townes (expressing normal human β^A^-globin), HbAS (heterozygous for β^A^- and β^S^-globin), and HbSS (homozygous for β^S^-globin) mice and in C57BL/6 mice. All circulating RBC were biotinylated at time zero. Five µL blood samples were obtained by tail vein puncture at various time intervals as indicated and the percentages of biotin labeled and unlabeled RBC were measured by flow cytometry. The half-lives of RBC in HbSS-Townes mice were markedly shorter (~2.5 days) than that of HbAA-Townes mice (~16 days). RBC in the HbAS-Townes mice had a half-life that was intermediate (~11 days) between AA and SS mice. The mean RBC half-life in normal C57BL/6 mice was 24 days, which was 50% longer than HbAA-Townes mice. The chronic hemolysis in HbSS mice can also be seen when measuring expired CO (Figure 2). The heme released from Hb during hemolysis eventually reaches tissues where the heme is degraded by HO, releasing CO that can be carried by Hb to the lungs and expired. Normal C57BL/6 mice release ~1 nmol/h/g of CO. When C57BL/6 mice are injected with phenylhydrazine (PHZ) to induce massive hemolysis, the expired CO goes to ~5 nmols/h/g. Similarly, HbAA mice expire ~1 nmols/h/g of CO and the HbSS mice expire 6–7nmols/h/g of CO, which is indicative of the chronic hemolysis in the HbSS mice. CO expiration in the HbAS mice is similar to HbAA mice, suggesting measurement of expired CO may not be as sensitive in determining hemolytic rates as measurement of RBC turnover. However, the elevated levels of CO in expired breath are consistent with HO catabolism of heme in states of excessive hemolysis.

**Figure 3:**
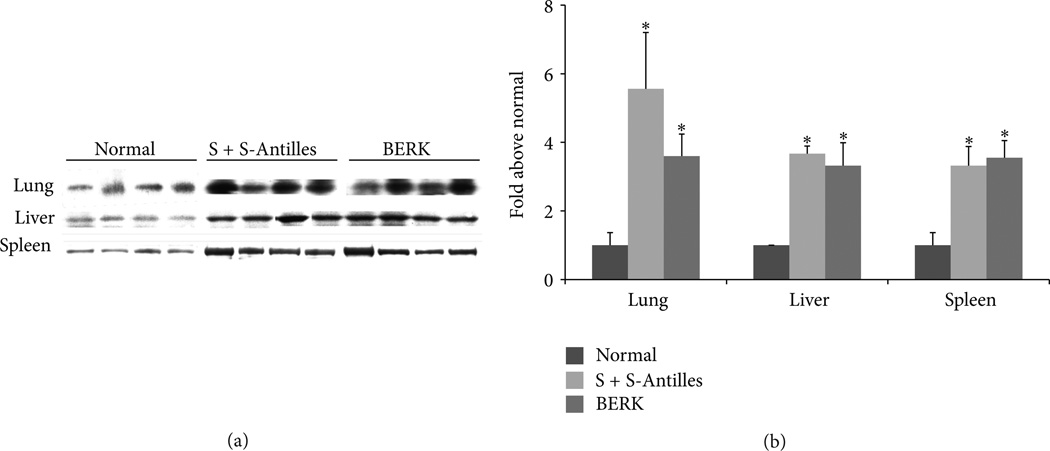
HO-1 expression is elevated in the organs of sickle mice. Western blots for HO-1 were performed on organ homogenates (1 µg of homogenate DNA per lane) from lungs, livers, and spleens of untreated normal mice and S + S-Antilles, and BERK sickle mice. The 32 kD HO-1 bands are shown for each organ and each mouse (a). The mean HO-1 band intensities (*n* = 4) ± SD are expressed as fold above normal control mice (b). *P < 0.05 normal versus sickle. Figure derived from [65] American Society for Clinical Investigation.

**Figure 4:**
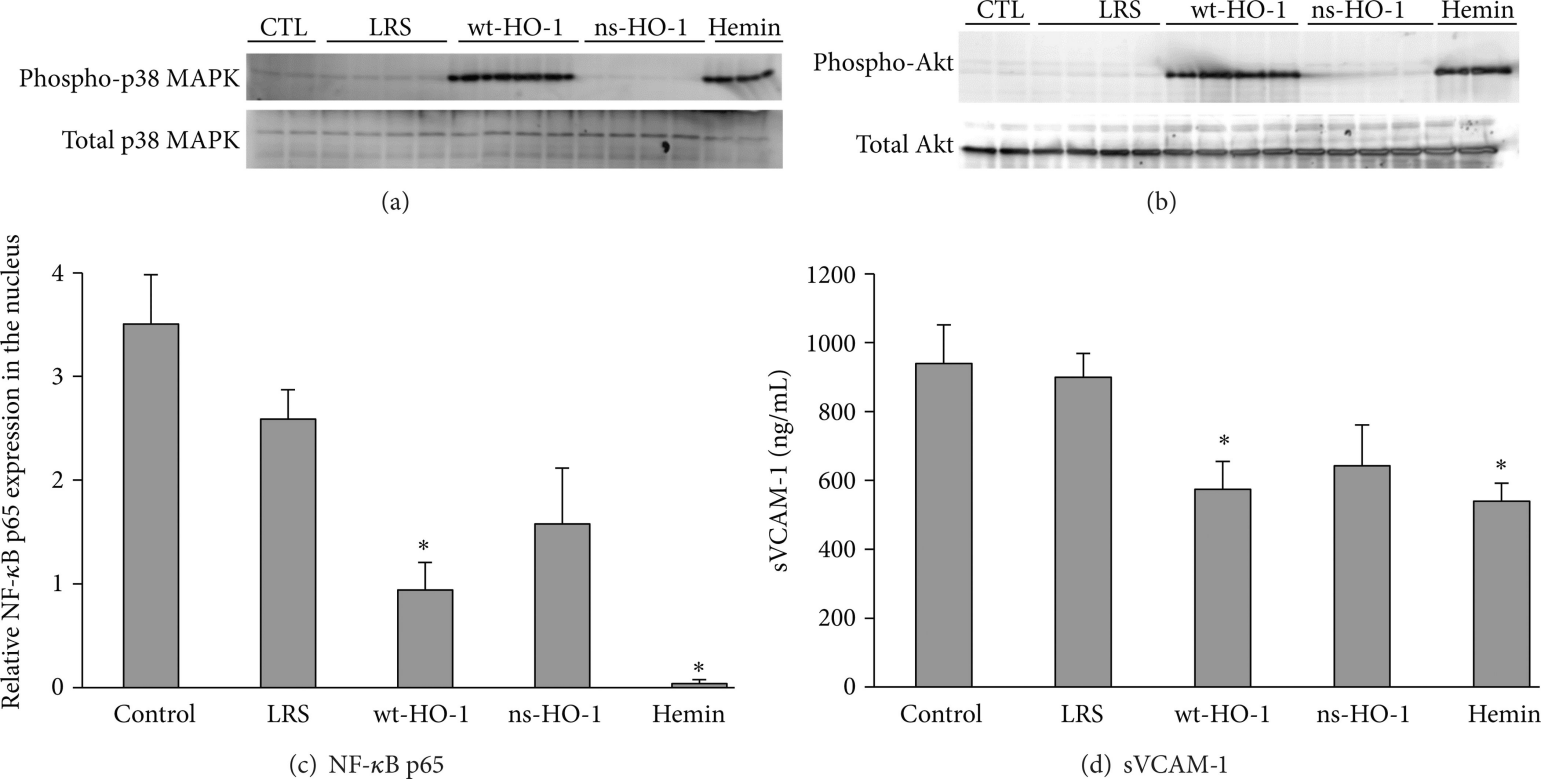
Cytoprotective pathways are activated in sickle mice overexpressing wild type (wt)-HO-1. Nuclear extracts were isolated from livers and 30 µ g of nuclear extract protein from each liver was run on a western blot and immunostained for phospho- and total p38 and Akt, and NF-κB p65. NF-κB p65 protein bands at 65 kDa were quantified by densitometry. Nuclear phospho-p38 MAPK (a) and phospho-Akt (b) were increased in mice 8 weeks after hydrodynamic infusion of *Sleeping Beauty* (*SB*)-wt-HO-1 DNA or after intraperitoneal injection of hemin chloride for 3 consecutive days, but not in control mice, given a hydrodynamic infusion of lactated Ringers solution (LRS) or *SB*-nonsense (ns)-HO-1 DNA. Total nuclear p38 MAPK (a) and Akt (b) were not different between treatment groups. NF-κB p65 was decreased in liver nuclear extracts of sickle mice injected with *SB*-wt-HO-1 or hemin, but not in control, LRS, or *SB*-ns-HO-1 treated mice (c). Serum levels of sVCAM-1 were measured by ELISA. Serum sVCAM-1 was lower in sickle mice injected with *SB*-wt-HO-1 DNA (*P* < 0.05) or hemin (*P* < 0.05), but not in mice infused with *SB*-ns-HO-1 DNA when compared to mice infused with LRS (d). Values are means ± SEM; *n* = 2–4 mice per treatment group; * *P* < 0.05 compared to LRS controls using one-way ANOVA. Figure derived from [66].

**Figure 5:**
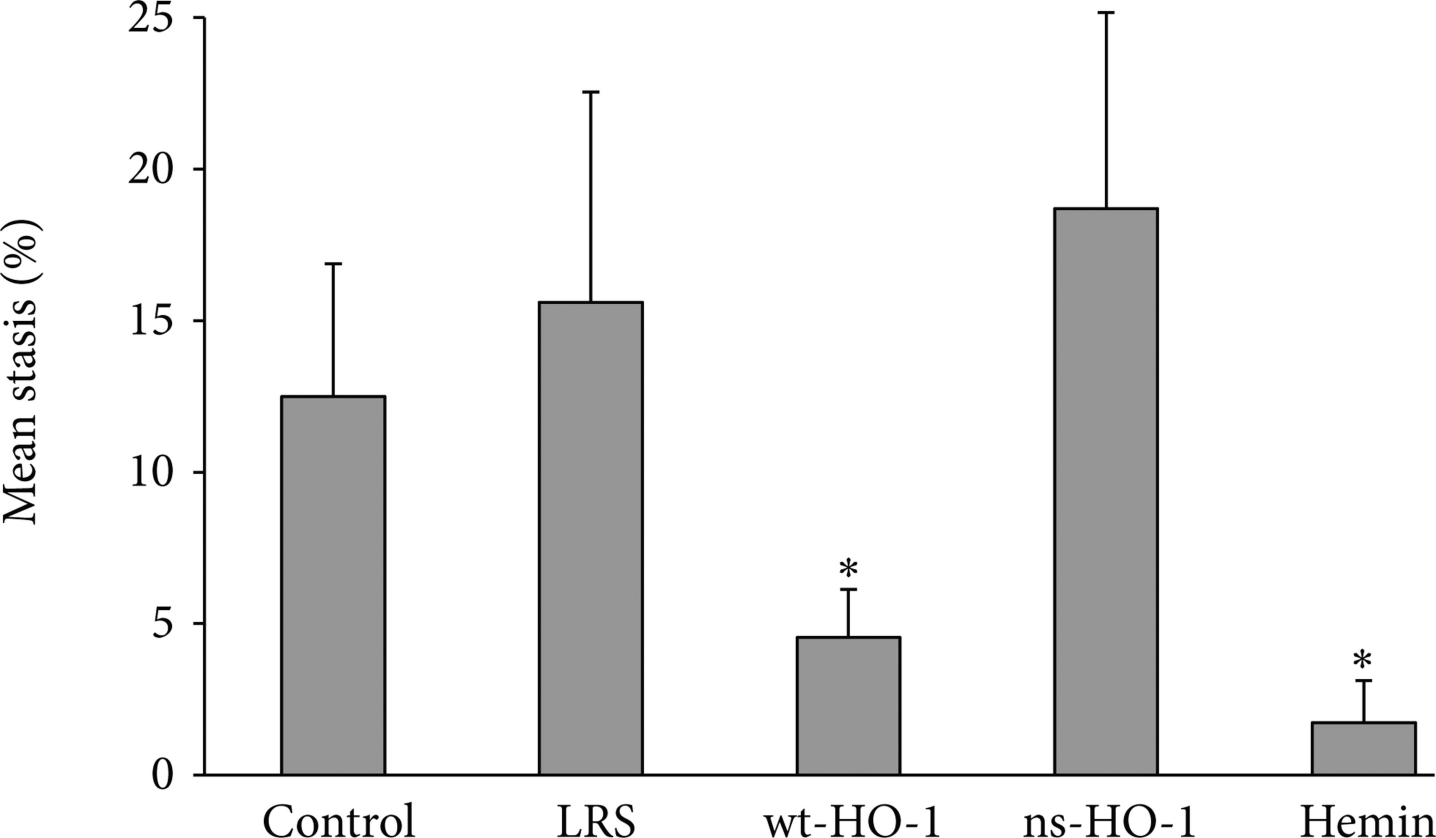
Vaso-occlusion (stasis) is inhibited in the skin of sickle mice 8 weeks after hydro dynamic infusion of liver-directed *SB*-wt-HO-1 or 24 h after 3 days of intraperitoneal hemin pretreatment (40 µmols/kg) compared to control mice or mice given hydrodynamic infusions of LRS or enzymatically inactive ns-HO-1. Vascular stasis was measured in a dorsal skin fold chamber (DSFC) model after H/R. At baseline in room air, the mice were placed under a microscope and flowing venules were selected inside the DSFC. The mice were then subjected to 1 h of hypoxia (7% O_2_/93% N_2_) followed by 1h of reoxygenation in room air. After 1h of reoxygenation, the same venules were reexamined for blood flow. The number of static venules exhibiting no blood flow was counted and expressed as a percentage of the total number of venules examined. There were 7 mice and 403 venules in the control group, 5 mice and 243 venules in the LRS group, 5 mice and 347 venules in the wt-HO-1 group, 6 mice and 325 venules in the ns-HO-1 group, and 5 mice and 227 venules in the hemin group. There was a minimum of 26 venules per mouse. Values are mean % stasis ± SEM. The proportions of venules exhibiting stasis in each treatment group were compared using a *z*-test; *P < 0.05 compared to LRS controls. Figure derived from [66].

Counteracting the toxic effects of hemolysis is the upregulation of HO-1 in tissues of transgenic sickle mice (Figure 3) and human sickle patients [65, 67]. Further increases in HO-1 inhibit vascular inflammation and vaso-occlusion in mouse models of sickle cell disease [65, 66]. Transgenic sickle mice with additional 3–5 fold overexpression of wild type (wt)-HO-1 in the liver using a *Sleeping Beauty* transposon system have activated nuclear phospho-p38 MAPK and phospho-Akt (Figures 4(a) and 4(b)), decreased nuclear expression of NF-κB p65 (Figure 4(c)), and decreased soluble vascular cell adhesion molecule-1 (sVCAM-1) in serum (Figure 4(d)) [66]. Pretreatment of sickle mice for 3 consecutive days with hemin (40 µmols/kg) intraperitoneally induced HO-1 in mouse tissues and mimicked the effects of wt-HO-1 overexpression via the *Sleeping Beauty* transposon system (Figures 4(a)−4(d)). Hypoxia-reoxygenation (H/R)-induced vaso-occlusion (stasis), a characteristic of sickle, but not normal mice, is inhibited in subcutaneous skin of sickle mice despite the absence of the HO-1 transgene in the skin suggesting distal effects of HO activity in the liver on the vasculature (Figure 5) [66]. No protective effects are seen in sickle mice overexpressing a nonsense rat *hmox-1* gene (ns-HO-1) that encodes carboxy-truncated HO-1 with little or no enzyme activity [66]. As previously shown [65], mice pretreated for 3 days with hemin intraperitoneally to induce HO-1 have significantly lower stasis (2.8%) than mice pretreated with lactated Ringers solution (LRS) (*P* < 0.006) [66]. The cytoprotective effects of HO-1 overexpression in transgenic sickle cell mice are mimicked by administration of inhaled CO or biliverdin [65].

In sickle mice and patients in steady state, hemolysis is in balance with HO activity; a biologic set point with just enough HO-1 induction to counterbalance hemolysis. However, hyperhemolysis can occur during vasoocclusive crises in patients with sickle cell disease [68], suggesting an increased rate of intravascular hemolysis may tip a sickle patients balance from steady state to vasoocclusive crisis. Vaso-occlusion is an inflammatory adhesion driven process [14, 65, 69]. Vaso-occlusion can be induced in transgenic sickle mice by exposing the mice to H/R or by infusing a bolus of Hb or hemin to simulate a sudden increase in intravascular hemolysis [14, 65, 69]. Any of these insults (H/R, Hb, and hemin) leads to stasis of blood flow in the postcapillary venules [14, 65, 69]. This vaso-occlusion can be blocked by inhibiting TLR4 signaling [14]; supporting the concept that hemin released from Hb during hemolysis promotes vaso-occlusion through hemin-induced TLR4 signaling and subsequent inflammatory response.

## 9. Potential Therapies for Heme-Induced Inflammation and Vascular Activation

The most effective strategy to avert heme-induced inflammation and vascular activation is to prevent or inhibit hemolysis. In sickle cell disease there is much effort underway to develop drugs to induce fetal Hb expression in RBC because of its ability to inhibit the polymerization of HbS and subsequent hemolysis. In lieu of effective strategies to stop hemolysis, the clinician must deal with the consequences of hemolysis. Infusions of supplemental haptoglobin and/or hemopexin are under consideration [7]. Other options include pharmacologic induction of the HO-1/ferritin system or inhibition of TLR4 signaling. This could possibly be accomplished by the administration of biliverdin [47, 70, 71], inhaled CO, CO-releasing molecules or CO bound to pegylated Hb [36, 43, 65, 72].

## 10. Conclusions

The release of free heme during hemolysis is injurious to cells. Extraordinary arrays of plasma and cellular defenses have evolved to protect cells from free heme. Failure to adequately protect against free heme can result in damage to the vasculature. This damage can be stopped by a robust anti-oxidative and anti-inflammatory response by vascular cells. With our current knowledge and understanding of these pathways, it may be possible to intervene clinically to bolster the cytoprotective responses and prevent much of the vascular damage associated with hemolysis.

## Abbreviations

Hb: Hemoglobin
RBC: Red blood cells
ROS: Reactive oxygen species
MetHb: Ferric hemoglobin
TLR4: Toll-like receptor-4
DAMP: Damage-associated molecular pattern
LPS: Lipopolysaccharide
TNF-α: Tumor necrosis factor-alpha
WPB: Weibel-Palade Body
NF-κB: Nuclear factor-kappa B
Nox: NADPH oxidase
HO: Heme oxygenase
MAPK: Mitogen-activated protein kinase
PKC: Protein kinase C
PI3K: Phosphatidylinositol 3-kinase
Akt: Protein kinase B
AP-1: Activator protein-1
Nrf2: Nuclear factor-erythroid 2 p45-related factor 2
BVR: Biliverdin reductase
Bach1: BTB and CNC homologue 1
Keap1: Kelch-like ECH-associated protein 1
ARE: Antioxidant response elements
MARE: Maf recognition element
FHC: Ferritin heavy chain
Ppm: Parts per million
HIF-1α: Hypoxia-inducible factor-1-alpha
eNOS: Endothelial nitric oxide synthase
IRP: Iron regulatory protein
IRE: Iron response element
MPTP: 1-Methyl-4-phenyl-1,2,3,6-tetrahydropyridine
DAXX: Death domain-associated protein
PHZ: Phenylhydrazine
Wt: Wild type
sVCAM-1: Soluble vascular cell adhesion molecule-1
H/R: Hypoxia-reoxygenation
Ns: Nonsense
LRS: Lactated Ringer’s solution
